# Supplementary material for: Identification of Freezing-Responsive microRNAs and Their Targets in Chinese Jujube by Small RNA and Degradome Sequencing
Source: Int J Mol Sci. 2026 Sep 4;27(17):7912. doi: 10.3390/ijms27177912 (PMC13566890; doi:10.3390/ijms27177912)
Supplement: Supplementary file 1 [file ijms-27-07912-s001.zip › Supplementary Material S2/novel_miR_140.pdf]

Provisional ID	:	novel_miR_140
Score total	:	2664.8
Score for star read(s)	:	3.9
Score for read counts	:	2656.8
Score for mfe	:	2.5
Score for randfold	:	1.6
Score for cons. seed	:	
Total read count	:	5223
Mature read count	:	5163
Loop read count	:	33
Star read count	:	27

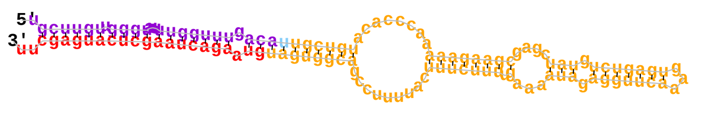

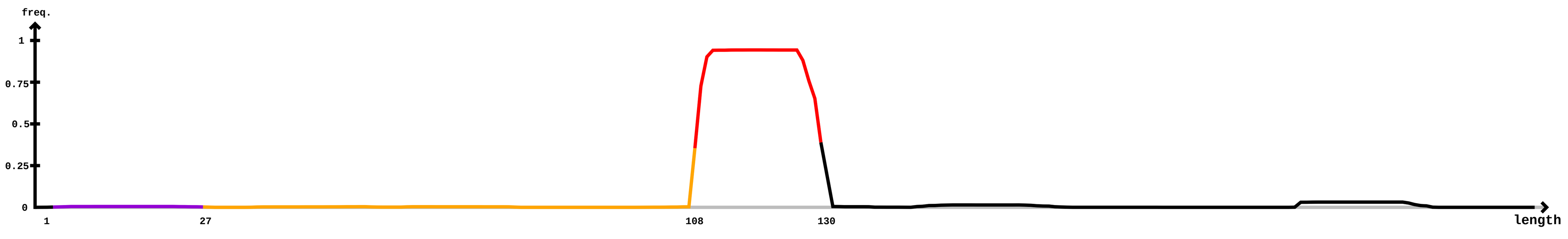

Star **Mature**

```
5'- augcuuguuggguauuuugguuugacauugcuguacacccaaaaagaagcgagcuaugucugagugaaacuuggagauaaaaguuuucuuucauuuuccgacggugauguaagacuaagcucaugagcuuauuauccuaggucgaaacaaguugauaggauccccuuuuuugggucccc augucccucccauguggugacgugggggcauaaaaaaggaaagagagggaugguguuucucucgcuuuuag  -3'  obs
    augcuuguuggguauuuugguuugacauugcuguacacccaaaaagaagcgagcuaugucugagugaaacuuggagauaaaaguuuucuuucauuuuccgacggugauguaagacuaagcucaugagcuuauuauccuaggucgaaacaaguugauaggauccccuuuuuugggucccc augucccucccauguggugacgugggggcauaaaaaaggaaagagagggaugguguuucucucgcuuuuag        exp
    ..((((((.((((...(((((((.((((((((((.......((((((((....(((.(((((((...))))))).)))...).))))))).........))))))))).))))))))))))))))).....(((((..(((((......))))))))))..(((((((((.((((((((((((((.......)).)))))))))))).)))))))))((((.((((((.......)))))).))))...   reads  mm  sample
    augcuugu uggguCuuuugguuu.......   1  1  JCK-1
    augcuuguuggguauuNgguuugaca.......   1  1  LT-2
    augcuuguuggguCuuuugguuugaca.......   1  1  JCK-1
    .ugcuuguuggguauuuugguA.......   1  1  LT-3
    .ugcuuguuggguCuuuugguuugac.......   1  1  JT-3
    .ugcuuguuggguCuuuugguuugaca.......   1  1  JT-2
    .ugcuuguuggguauuuugguuugCca.......   1  1  JCK-1
    .ugcuuguuggguauuuuggCuugaca.......   1  1  LT-3
    .ugcuuguuggguCuuuugguuugaca.......   1  1  LCK-3
    ..gNuuguuggguauuuugguu.......   1  1  JT-1
    ..gcuuguuggguauuuugguA.......   1  1  LT-1
    ..gcuuguuggguauuuugguuuUaca.......   1  1  JT-3
    ..gcuuguuggguauuuugguuugacC.......   1  1  LT-2
    ..Ucuuguuggguauuuugguuugaca.......   1  1  LT-3
    ...cuuguuggguCuuuugguu.......   1  1  LT-2
    ...cuuguuggguCuuuugguuug.......   1  1  LT-2
    ...cuuguuggguauuuugguuugCc.......   1  1  LT-1
    ...cuuguuggguCuuuugguuugac.......   1  1  LCK-3
    ...cuuguuggguCuuuugguuugac.......   1  1  LT-2
    ...cCuguuggguauuuugguuugaca.......   1  1  LT-1
    ....uuguuggguauuuuggGuug.......   3  1  LT-2
    ....uuguuggguauuuCgguuuga.......   1  1  LT-3
    ....uuguuggguauuuugUuuugac.......   1  1  LT-2
    ....uuguuggguauuuugguuugacC.......   1  1  LT-1
    ........uggguauuuugguuugCca.......   1  1  JCK-2
    ..................................acacccaaaaagaagcgagc.......   1  0  LT-3
    ..................................acacccaaaaagaagcgagc.......   1  0  LT-1
    ...................................cacccaaaaagaagcgagc.......   3  0  LCK-3
    ...................................cacccaaaaagaagcgagc.......   1  0  LT-3
    ...................................cacccaaaaagaagcgagcu.......   4  0  LT-1
    ....................................acccaaaaagaagcgagc.......   2  0  LT-3
    ....................................acccaaaaagaagcgagcua.......   1  0  LT-3
    ......................................ccaaaaagaagcgagcua.......   1  0  LT-3
```

Star **Mature**

augcuuguugggua uuuugguuugacauugcuguacacccaaaaagaagcgagcuaugucugagugaaacuuggagauaaaaguuuucuuucauuuuccgacggugauguaagacuaagcucaugagcuuauuauccuaggucgaaacaaguugauaggauccccuuuuuugggucccca ugucccucccauguggugacguggggg cauaaaaaaggaaagagagggaugguguuucucucgcuuuuag

Read	Count	Mismatches	Sample
aagaagcgagcuaAgucugagu	1	1	LCK-2
agcgagcuaugucugagugaaacuuggaga	1	0	LCK-1
cgagcuaugucugagugaaacuug	1	0	LT-3
gagcuaCgucugagugaaacuuggagaua	2	1	LT-2
gcuaCgucugagugaaacuuggagaua	2	1	LT-2
ugagugaaacuuggagau	8	0	LCK-3
ugagugaaacuuggagau	1	0	LT-2
gagugaaacuuggagaua	3	0	LT-1
gacggugaCguaagacuaagcuc	1	1	LT-2
acggugaCguaagacuaagcucaugagcuu	1	1	JCK-2
cggugaCguaagacuaagc	1	1	LT-2
cggugaCguaagacuaagcucauga	1	1	LCK-3
gugaCguaagacuaagcuc	1	1	LT-2
ugaCguaagacuaagcucauga	1	1	LT-2
ugaCguaagacuaagcucaugagcuu	2	1	LT-2
gaCguaagacuaagcucaugag	1	1	LT-3
aNguaagacuaagcucauga	1	1	JCK-1
aCguaagacuaagcucaugag	1	1	LCK-2
aCguaagacuaagcucaugagc	1	1	JCK-1
aNguaagacuaagcucaugagcu	1	1	LT-3
aCguaagacuaagcucaugagcu	2	1	LT-2
Cguaagacuaagcucaugagcuu	1	1	LT-3
guCagacuaagcucauga	1	1	LT-2
Uuaagacuaagcucauga	2	1	LT-2
guaagacuaagcucauga	80	0	LCK-2
guaagacuaagcucauga	2	0	JCK-2
guaagacuaagcucauga	15	0	LT-3
guaagacuaagcucauga	4	0	JCK-3
guNagacuaagcucauga	1	1	LT-2
guaagacuaagcucauga	22	0	LCK-3
guaagacuaagcucauga	12	0	JT-1
guaagacuaagcucauga	1	0	JCK-1
guaagacuaagcucauga	35	0	JT-2
guaagacuaagcucauga	29	0	LT-2
guaagacuaagcucauga	47	0	JT-3
Auaagacuaagcucauga	1	1	LCK-2
guaagacuaagcucauga	15	0	LT-1
guaagacuaagcucauga	71	0	LCK-1
guaagacuaagcucaugag	32	0	JT-3
guaagacuaagcucNugag	1	1	LCK-1
guaagacuaagcucaugag	39	0	LT-2
guaagacuaagcucaugag	15	0	LCK-2
guaagacuaagcucaugag	22	0	JT-2
guaagGcuaagcucaugag	1	1	JCK-2
guaagacuaagcucaugag	3	0	JCK-3
guaagacuaagcucaugag	26	0	LCK-3
guaagacuaagcucaugag	18	0	LT-1
guaagacuaagcucaugag	4	0	JCK-2
guaagacuaagcucaugag	5	0	JCK-1
guaagacuaagcucaugag	14	0	LCK-1
guCagacuaagcucaugag	2	1	LT-2
guaagacuaagcucaugag	33	0	LT-3
guaagacuaagcucaugagc	10	0	LCK-3
guaagacuaagcucaugagc	27	0	LT-3
guaagacuaagcucaugagc	7	0	JT-3
guaagacuaagcucaugagc	7	0	JT-2
guaagacuaagcucaugagc	13	0	JCK-3
guaagacuaagcucaugagc	9	0	JCK-1
guaagacuaagcucaugagc	22	0	LT-1
guaagacuaagcucaugagc	20	0	JCK-2
guaagacuaagcucaugagc	25	0	LT-2
guaagacuaagUucaugagcu	1	1	JT-3
Uuaagacuaagcucaugagcu	1	1	LT-3
guaagacuaagcucaugagcC	1	1	LT-3
guaagacuaagcucaugagcu	70	0	LT-3
guaagacuaagcucaugagcu	72	0	LT-2
guaagacuaagcucaugagcu	9	0	JT-3
guaagacuaagcucaugagcu	13	0	JCK-1
guaagacuaagcucaugagcu	45	0	LCK-3
guaagacuaagcucaugagcu	55	0	LT-1

Star … **Mature**

augcuuguugggguauuuugguuugacauugcuguacacccaaaaagaagcgagcuaugucugagugaaacuuggagauaaaaguuuucuuucauuuuccgacggugauguaagacuaagcucaugagcuuauuauccuaggucgaaacaaguugauaggauccccuuuuuugggucccaugucccucccaugugggugacgugggggcauaaaaaaggaaagagagggaugguguuucucucgcuuuuag

Read			
guaagacuaagcucaugagcu	15	0	JCK-3
guaagacuaagcucaugagcA	1	1	JCK-3
guaagacuaagcucaugagcu	72	0	LCK-1
guaagacuaagcucaugagcu	13	0	JCK-2
guaagacuaagcucaugagcu	8	0	JT-2
guaagacuaagcucaugagAu	1	1	JCK-3
guaagacuaagcucaugagcu	69	0	LCK-2
guaagacuaagcucaugagcuu	39	0	JT-1
guaagacuaagcucaugagcuu	18	0	JCK-1
guCagacuaagcucaugagcuu	1	1	JT-3
guaagacuaagcucCugagcuu	1	1	LCK-2
guaagacuaagcucaugagcuu	23	0	JCK-3
guCagacuaagcucaugagcuu	1	1	LCK-2
guaagacuaagUucaugagcuu	1	1	JT-2
guCagacuaagcucaugagcuu	1	1	LT-2
guaagacuaagcucaugagcuu	37	0	JT-2
guaagacuaagUucaugagcuu	1	1	LT-1
guaagacuaagcucaugagcuu	101	0	LCK-1
guaagacuaagcucaugagcuu	64	0	LCK-3
guaagacuaagcucaugagcuu	66	0	JT-3
Auaagacuaagcucaugagcuu	1	1	LCK-1
guaagacuaagcucaugagcuu	123	0	LT-2
guaagacuaagcucaugagcuu	84	0	LCK-2
guaagacuaagcucaugagcuu	108	0	LT-3
guCagacuaagcucaugagcuu	1	1	JT-1
guaagacuaagcucaugagcuu	28	0	JCK-2
guaagacuaagcucaugCgcuu	1	1	LT-3
guaagacuaagcucaugagcuu	84	0	LT-1
guaagacuaagcucaugagcuuauu	3	0	LCK-3
guaagacuaagcucaugagcuuauuauccu	3	0	JCK-2
guaagacuaagcucaugagcuuauuauccu	2	0	JCK-1
guaagacuaagcucaugagcuuauuauccu	2	0	LCK-3
guaagacuaagcucaugagcuuauuauccu	1	0	LT-3
uaagacuaaUcucaugag	1	1	LCK-2
uaagacuaCgcucaugag	1	1	LCK-1
uaagacuaagcucaugag	58	0	LCK-2
uaagacuaagcucaugag	83	0	LCK-1
uaagacuaagcucaugag	38	0	JT-3
uaagacuaagcucaugag	3	0	JCK-3
uaagacuaagcucaugag	8	0	JCK-1
uaagacuaagcucaugag	25	0	JT-2
uaagacuaagcucaugag	41	0	LT-3
uaagacuaagcucaugag	5	0	JT-1
uaagacuaagcucauCag	1	1	LT-2
uaagacuaagcucaugag	6	0	JCK-2
uaagacuaagcucaugag	22	0	LT-1
uaagacuaagcucaugag	120	0	LT-2
uaagacuaagcucaugag	45	0	LCK-3
uaagacuaagcucaugagc	24	0	JCK-3
uaagacuaagcucaugagc	17	0	JCK-1
uaagCcuaagcucaugagc	1	1	LT-2
uaagacuaagcucaugagc	17	0	JT-3
uaagacuaagcucaugagc	28	0	LT-2
uaaUacuaagcucaugagc	1	1	LT-1
uaagacuaagcucaugagc	11	0	JT-1
Caagacuaagcucaugagc	1	1	JCK-1
uaagacuaagcucaugagc	46	0	LT-1
uaagacuaagcucaugagc	11	0	JT-2
uaagacuaagcucaugagc	36	0	JCK-2
uaagacuaagcucaugagc	14	0	LCK-3
uaagacuaagcucaugagc	45	0	LT-3
uaagacuaagcucaugagcu	16	0	JCK-3
uaagacuCagcucaugagcu	1	1	LT-1
uaagacuaagcucaugagcC	1	1	LCK-3
uaagacuaagcucaugagcA	1	1	LCK-3
uaagacuaagcucaugagcu	12	0	JCK-1
uaagacuaagcucaugagcu	8	0	JT-2
uaagacuaagcucCugagcu	1	1	LCK-3
uaaAacuaagcucaugagcu	1	1	JCK-3
uaagCcuaagcucaugagcu	1	1	LT-2

Star **Mature**

augcuuguuggguauuuuugguuugacauugcuguacacccaaaaagaagcgagcuaugucugagugaaacuuggagauaaaaguuuucuuucauuuuccgacggugauguaagacuaagcucaugagcuuauuauccuaggucgaaacaaguugauaggaucccccuuuuuugggucccccaugucccucccauguggugacgugggggcauaaaaaaggaaagagagggaugguguuucucucgcuuuuag

...uaagCcuaagcucaugagcu...	1	1	JCK-3
...uaagacuaagcucaugagcA...	1	1	JCK-2
...uaagacuaagUucaugagcu...	1	1	LT-2
...uaagacuaagcucaugagcu...	24	0	JCK-2
...uaagacuaagcucaugagcu...	63	0	LCK-3
...uaagacuaagcucaugagcu...	89	0	LT-1
...uaagacuaagcucaugagcu...	18	0	JT-1
...uaagacuaagcucaugagcu...	5	0	JT-3
...uaagacuaagcucaugagcu...	105	0	LT-2
...uaagacuaagcucaugagcu...	76	0	LT-3
...uaagacuCagcucaugagcuu...	1	1	LT-2
...uaagacuaagcucaugagcuu...	75	0	JT-3
...uaagacuaagcucaugagcuu...	98	0	LT-2
...uaagacuaagcucaugagcuu...	41	0	JCK-3
...uaagacuaagcucaugagcuu...	56	0	LCK-2
...uaagacuaagcucaugagcuu...	99	0	LT-1
...uCagacuaagcucaugagcuu...	1	1	LCK-2
...uaagacuaagcucaugagcuu...	119	0	LT-3
...Caagacuaagcucaugagcuu...	1	1	LT-1
...uaagacuaagcucaugagcuu...	81	0	JT-1
...uaagacuaagcucaugagcuu...	39	0	JT-2
...uaagacuaagcucaugagcuu...	84	0	LCK-1
...uaagacuaagAucaugagcuu...	1	1	LCK-2
...uaagacuaagcucaugagcuu...	52	0	JCK-2
...uaagacuaagcucaugaAcuu...	1	1	LCK-2
...uaCgacuaagcucaugagcuu...	1	1	JT-1
...uaagacuaagUucaugagcuu...	1	1	JT-3
...uaagacuaagcucaugagcuu...	107	0	LCK-3
...uaagacuaagcucaNgagcuu...	1	1	LCK-1
...uaagacuGagcucaugagcuu...	1	1	JCK-3
...uaagacuaagcucaugagcuu...	48	0	JCK-1
...uaagacuaagcucaugagAuu...	1	1	LCK-2
...uaagacuaagcucaugagcuua...	4	0	LT-1
...uaagacuaagcucaugagcuua...	1	0	LT-3
...uaagacuaagcucaugagcuuauu...	1	0	LT-3
...uaagacuaagcucaugagcuuauu...	2	0	JCK-2
...aagacuaagcucaugagc...	23	0	JCK-1
...aagacuaagcucaugagc...	32	0	LT-3
...aagacuaagcucaugagc...	31	0	JCK-3
...aagacuaagcucaugCgc...	1	1	LT-1
...aagacuaagcucaugagc...	17	0	LCK-3
...aagacuaagcucaugagc...	42	0	JCK-2
...aCgacuaagcucaugagc...	1	1	LT-3
...aagacuaagcucCugagc...	1	1	JCK-1
...aagacuaagcucaugagc...	7	0	JT-3
...aagacuaagcucaugagc...	12	0	LT-2
...aagacuaagcucaugagc...	25	0	LT-1
...aagacuaagcucaugagcu...	30	0	JT-1
...aagacuaagcucaugagcu...	99	0	LCK-3
...Uagacuaagcucaugagcu...	1	1	LT-3
...aagacuaagcucaugagcu...	42	0	LT-2
...aagacuaagcucauUagcu...	1	1	LT-1
...aagacuaagcucaugagcu...	70	0	LT-1
...aagacuaagcucaugagcu...	17	0	JCK-3
...aagacuaagcucaugagcu...	76	0	LT-3
...aagacuCagcucaugagcu...	1	1	LT-2
...aagacuaagcucaugagcu...	25	0	JT-2
...aagacuaagcuNaugagcu...	1	1	JT-1
...aagacuaagcucaugagcu...	24	0	JCK-2
...aagacuaagcucaugagcA...	1	1	LCK-3
...aagacuaagcucaugagcu...	26	0	JT-3
...aagacuaagcucCugagcu...	1	1	JCK-3
...aagacuaagcucaugagcu...	10	0	JCK-1
...aagacuaCgcucaugagcu...	1	1	LT-1
...aagacuaagcucaugagcA...	1	1	LT-2
...aagacuaagcucaugagcuu...	10	0	JT-2
...aagacuaagcucaugagcuu...	12	0	JCK-3
...aagacuaagcucaugagcuu...	3	0	JT-1
...aagacuaagcucCugagcuu...	1	1	JT-3
...aagacuaagcucaugagcuu...	31	0	LT-1

Star **Mature**

augcuuguuggguauuuugguuugacauugcuguacacccaaaaagaagcgagcuaugucugagugaaacuuggagauaaaaguuuucuuucauuuuccgacggugauguaagacuaagcucaugagcuuauuauccuaggucgaaacaaguugauaggauccccuuuuuuggguccccauguccccucccauguggugacgugggggcauaaaaaaggaaagagagggaugguguuucucucgcuuuuag

aagacuaagcucaugagcuu	11	0	JT-3
aagacuaagcucaugagcuu	53	0	LT-3
aagacuaagcucaugagcuu	69	0	LT-2
aagacuaagcucaugagcuu	11	0	JCK-2
aagacuaagcucaugagcuu	33	0	LCK-2
aagacuCagcucaugagcuu	1	1	LT-3
aagacuaagcucaugagcuu	20	0	LCK-1
aagacuaagcucaugagcuu	16	0	JCK-1
aagacuaaUcucaugagcuu	5	1	LT-2
aagacuaagcucaugagcuu	64	0	LCK-3
aagacuaagUucaugagcuu	1	1	JCK-2
aagacuaagcucaugaUcuuauuaucc	1	1	JT-3
agacuaagcucaugagcA	1	1	LT-1
agacuaagcucaugagcu	2	0	JCK-1
agacuaagcucaugagcu	11	0	LCK-3
agacuaagcucaugagcA	1	1	LT-3
agacuaagcucaugagcu	5	0	JT-1
agacuaCgcucaugagcu	1	1	LT-2
agacuaagcucaugagcu	39	0	LT-2
agacuaagcucaugagcu	4	0	JCK-2
agacuaagcucaugagcu	2	0	JCK-3
agacuaagcucaugagcu	15	0	LT-1
agacuaagcucaugagcu	36	0	LT-3
agacuaagcucaugGgcu	1	1	JCK-1
agacuaagcucaugagcu	7	0	JT-2
agacuaagcucaugagcu	21	0	JT-3
agacuaagcucaugagcuA	1	1	LT-1
agacuaagcucaugagcuu	2	0	JCK-2
agacuaagcucaugagcuu	10	0	LT-3
agacuaagcucaugagcuA	3	1	LT-2
agacuaagcucaugagcuu	6	0	JT-2
agacuaagcucaugagcuu	1	0	JCK-1
agacuaagcucaugagcuu	17	0	JT-1
agacuaagUucaugagcuu	1	1	JT-2
agacuaagcucaugagcuu	7	0	LT-2
agacuaagcucaugagcuu	4	0	LT-1
agacuaagcucaugagcuu	4	0	LCK-3
agacuaagcucaugagcuA	1	1	LT-3
agacuaagcucaugagcuA	3	1	LCK-3
agacuaagcucaugagcuu	9	0	JT-3
agacuaagcucaugagcuuauuauccu	1	0	JCK-2
gacuaagcucaugagcuu	2	0	LT-3
gacuaagcucaugagcuuauuauccu	1	0	LT-3
cuaagcucaugagcuuaG	2	1	JT-2
cuaagcucaugagcuuauuauccu	1	0	JCK-3
cuaagcucaugagcuuauuauccu	2	0	JCK-2
cuaagcucaugagcuuauuauccuaggucg	1	0	LT-2
uaagcucaugagcuuauuauccuagguc	1	0	JCK-2
agNucaugagcuuauuauccuagguc	1	1	LT-2
auNagcuuauuauccuag	1	1	LT-2
auccuaggucgCaacaaguuga	1	1	LT-3
aacaaguugauaggaucc	4	0	LT-2
aacaaguugauaggaucc	1	0	LT-1
aacaaguugauaggauccc	2	0	LT-1
aacaaguugauaggauccc	3	0	LT-3
aacaaguugauaggaucccc	5	0	JT-3
aacaaguugauaggaucccc	2	0	JT-2
aacaaguugauaggaucccc	1	0	LT-2
aacaaguugauaggaucccc	1	0	LT-3
aacaaguugauaggauccccuuu	3	0	LT-1
aacaaguugauaggauccccuuu	2	0	LT-3
acaaguugauaggauccc	1	0	LT-1
acaaguugauaggauccc	1	0	LT-3
acaaguugauaggaucccc	2	0	LT-1
acaaguugauaggauccccu	1	0	LT-1
acaaguugauaggauccccu	1	0	JCK-2
acaaguugauaggauccccuu	1	0	LT-1
acaaguugauaggauccccuuuu	1	0	LT-1
acaaguugauaggauccccuuuuu	1	0	LT-3
caaguugauaggaucccc	1	0	LT-2

Star **Mature**

augcuuguugggguauuuugguuugacauugcuguacacccaaaaagaagcgagcuaugucugagugaaacuuggagauaaaaguuuucuuucauuuuccgacggugauguaagacuaagcucaugagcuuauuauccuaggucgaaacaaguugauaggauccccuuuuuugggucccaugucccucccauguggugacguggggcauaaaaaaggaaagagagggaugguguuucucucgcuuuuag

caaguugauaAgaucccc	1	1	LT-1
caaguugauaggaucccc	2	0	LT-3
caaguugauaggauccccu	2	0	LT-3
caaguugauaggauccccu	4	0	LT-1
caaguugauaggauccccuu	1	0	LT-3
caaguugauaggauccccuu	1	0	LT-1
caaguugauaggauccccuuu	3	0	LCK-3
caaguugauaggauccccuuu	3	0	LT-1
caaguugauaggauccccuuuuu	2	0	LT-3
caaguugauaggauccccuuuuuu	3	0	LCK-3
aaguugauaggauccccuuu	1	0	LT-3
aaguugauaggauccccuuuuu	1	0	LT-1
aguugauaggauccccuuu	2	0	LT-3
aguugauaggauccccuuu	5	0	LT-2
aguugauaggauccccuuuu	3	0	LCK-3
aguugauaggauccccuuuu	1	0	LT-1
guugauaggauccccuuu	2	0	LT-3
guugauaggauccccuuuu	1	0	LT-3
guugauaggauccccuuuuuu	1	0	LT-1
uugauaggauccccuuuu	1	0	LT-3
uugauaggauccccuuuu	1	0	LT-1
uugauaggauccccuuuuu	2	0	LT-3
cccuuuuuuCgguccccaugucccc	1	1	LCK-3
aAaaaaaaggaaagagagggau	4	1	LT-2
uaaaaaaggaaagagagg	1	0	JCK-2
uaaaaaaggaaagagagg	7	0	JT-2
uaaaaaaggaaagagagg	2	0	LT-3
uaaaaaaggaaagagagg	1	0	JCK-3
uaaaaaaggaaagagagg	5	0	JT-3
uaaaaaaggaaagagagg	4	0	LCK-3
uaaaaaaggaaagagagg	7	0	LT-1
uaaaaaaCgaaagagagg	2	1	LT-3
uaaaaCaggaaagagagg	1	1	JT-2
uaaaaaaggaaagagaggg	16	0	JT-1
uaaaaaaggaaagagaggg	10	0	JT-2
uaaaaaaggaaagagaggg	11	0	JT-3
uaaaaaaggaaagagaggg	3	0	LT-3
uaCaaaaggaaagagaggg	1	1	LT-2
uCaaaaaggaaagagaggg	1	1	LT-1
uaaaaaaggaaagagaggg	6	0	LT-1
uaaaaaaggaaagagaggg	3	0	LT-2
uaaaaaaggaaagagaggga	2	0	LT-3
Aaaaaaaggaaagagaggga	1	1	LT-3
uaaaaaaggaaagagaggga	3	0	JCK-2
uaaaaaaggaaagagaggga	2	0	LT-1
uaaaaaaggaaagagaggga	12	0	JT-3
uaaaaaaggaaagagaggga	2	0	LCK-3
uaaaaaaggaaagagaggga	10	0	JT-2
uaaaaaaggaaagagagggau	1	0	LT-2
uaaaaaaggaaagagagggau	6	0	LT-3
uaaaaaaggaaagagagggaug	10	0	LT-1
uaaaaaaggaaagagagggaug	5	0	LT-3
uaaaaaaggaaagagagggaug	22	0	JT-1
uaaaaaaggaaagagagggaug	2	0	LT-2
uaaaaaaggaaagagagggaug	1	0	JCK-2
uaaaaaaggaaagagagggaugg	1	0	JT-3
uaaaaaaggaaagagagggauNg	1	1	JT-2
uaaaaaaggaaagagagggaugg	1	0	JT-2
aCaaaaggaaagagagggaug	1	1	LT-1
aaaaaggaGagagaggga	1	1	LT-2
aaCaaggaaagagagggaug	1	1	JT-2
aaaCaggaaagagagggaugg	1	1	JT-1
aaaagAaaagagagggaugg	1	1	LT-1
